# Sclerosing Encapsulating Peritonitis: Abdominal Cocoon

**DOI:** 10.7759/cureus.34322

**Published:** 2023-01-29

**Authors:** Sho Fujiwara, Ryujiro Akaishi, Tomoki Yokosawa

**Affiliations:** 1 Surgery, Iwate Prefectural Ofunato Hospital, Ofunato, JPN; 2 Emergency and Critical Care Center, Iwate Prefectural Ofunato Hospital, Ofunato, JPN

**Keywords:** small bowel resection, resection, sclerosing encapsulating peritonitis, small-bowel obstruction, tuberculosis peritonitis, abdominal cocoon

## Abstract

Sclerosing encapsulating peritonitis is a rare chronic inflammatory condition often with unknown origins. We report a case of an abdominal cocoon or sclerosing encapsulating peritonitis, which was suspected to be a result of bowel obstruction. Tuberculosis peritonitis was also suspected. However, the exact diagnosis was unclear, and it was diagnosed as an idiopathic abdominal cocoon. The patient’s history is of clear relevance in this diagnosis, and this report will be of interest to clinicians attending to cases of bowel obstruction.

## Introduction

Sclerosing encapsulating peritonitis (SEP) is a rare disease of uncertain cause characterized by intestinal obstruction followed by encapsulation of the small intestine with a fibro-collagen membrane [1]. Various conditions, such as peritoneal dialysis, history of abdominal surgery, ventriculoperitoneal shunt, tuberculosis, sarcoidosis, protein S deficiency, usage of beta blockers, familial Mediterranean fever, and cirrhosis, cause it [1-3]. Preoperative diagnosis is often difficult. We must consider SEP as a differential diagnosis of unusual bowel obstruction.

## Case presentation

**Figure 1:**
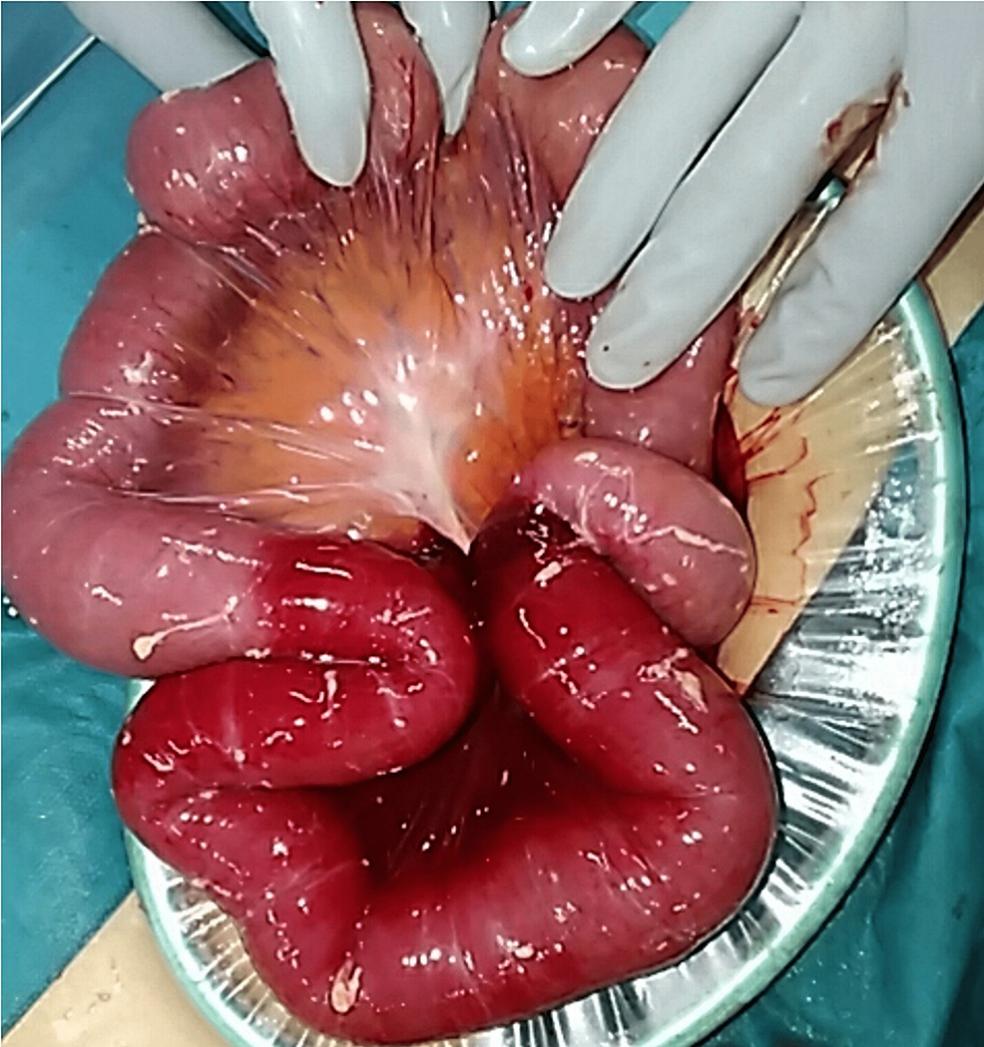
Operative finding of previous surgery He underwent release surgery without bowel resection for strangulated small bowel obstruction 35 days prior.

An 82-year-old man presented with a three-day history of abdominal distention and nausea. His family had a history of tuberculosis, and his past surgical history included strangulated small bowel obstruction 35 days prior. Then, he underwent release surgery without bowel resection (Figure 1).

**Figure 2:**
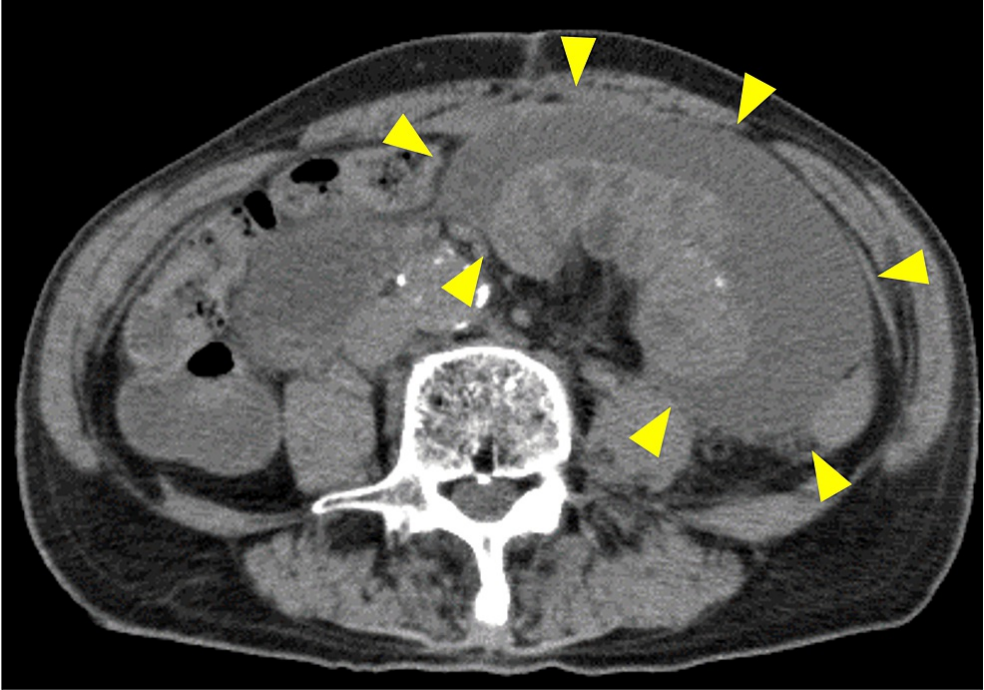
Abdominal computed tomography Abdominal CT showed a mass of jejunum in the encapsulated area with ascites and calcified nodules.

Abdominal ultrasound sonography revealed ascites around the small intestine in the encapsulated area. Abdominal computed tomography further showed a mass of jejunum in the encapsulated area with ascites and calcified nodules (Figure 2).

**Figure 3:**
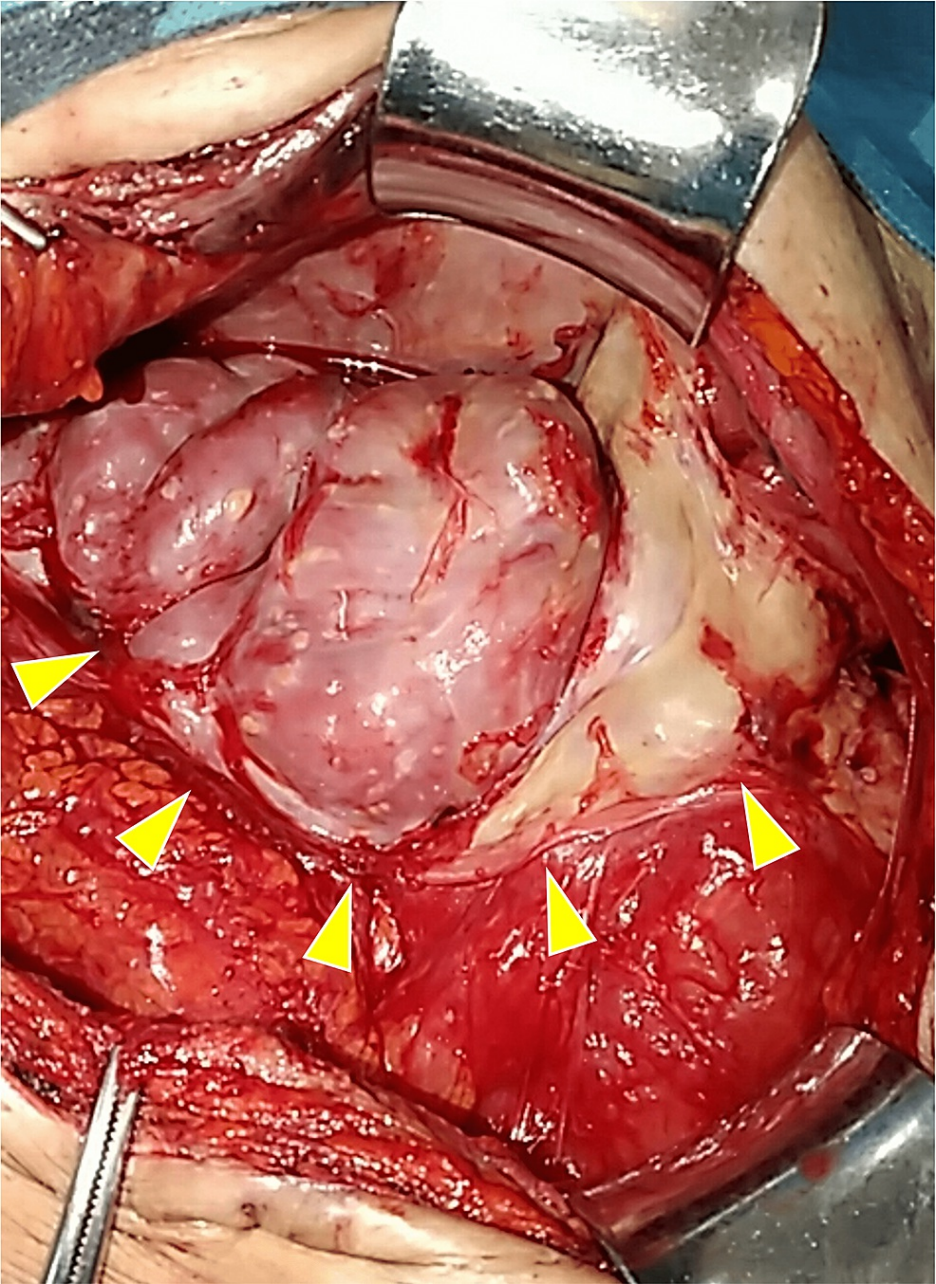
Operative finding The jejunum was enveloped in dense fibrous tissue with calcified nodules.

We aspirated ascites comprising serous blood. Cytology, culture, including Mycobacterium tuberculosis, and other ascites laboratory data did not indicate malignancy or tuberculosis. A blood test did not confirm tuberculosis nor other malignancy; interferon-gamma release assays, CA125, and other tumor markers were unremarkable. Then, serum albumin and C-reactive protein were 2.5 g/dL and 6.4mg/dL, respectively. The nasogastric tube did not improve the ileus, and he underwent surgery. The small intestine was enveloped in dense fibrous tissue with calcified nodules and became a contiguous mass like sclerosing encapsulating peritonitis (SEP), also known as an abdominal cocoon (Figure 3).

This section, which lacked peristalsis without signs of obstruction, was resected. Histopathological evaluation of the capsule, nodules, and resected bowel did not reveal an apparent cause of abdominal cocoon; chronic dense fibrous tissue without caseous granuloma. Thus, we diagnosed an idiopathic abdominal cocoon. While tuberculosis peritonitis was most likely, we did not confirm the cause of the abdominal cocoon.

## Discussion

SEP is a rare disease causing bowel obstruction. Our radiographic findings are similar in tuberculosis, peritoneal carcinomatosis, Crohn's disease, and sarcoidosis [4]. Thus, in our case, tuberculous peritonitis is the most likely cause of SEP. However, in this case, we could not confirm the tuberculous peritonitis. The sensitivity of histology for diagnosing tuberculous peritonitis is 93% [5]. Inflammation of obstructed jejunum was suspected of causing SEP. Surgical treatment consists of careful dissection, excision of the thick sac, and release of the small intestine [1]. However, bowel resection is required when stricture or functional bowel obstruction is accompanied, considering the short bowel syndrome. Thus, we must consider idiopathic SEP as the differential diagnosis in bowel obstruction with encapsulation, particularly for patients with calcification or a history of tuberculosis. Despite no evidence of SEP in prior surgery within approximately 30 days, continuous inflammation with mononuclear cells by strangulated bowel obstruction could be suspected to induce SEP [6].

## Conclusions

SEP is a rare condition that can present in patients with bowel obstruction. Even if past surgical history of bowel obstruction is within about 30 days, we must consider SEP as a differential diagnosis.
